# Supplementary material for: Childhood gut microbiome is linked to internalizing symptoms at school age via the functional connectome
Source: Nat Commun. 2025 Oct 30;16:9359. doi: 10.1038/s41467-025-64988-6 (PMC12575631; doi:10.1038/s41467-025-64988-6)
Supplement: Supplementary file 1 — Supplementary Information [file 41467_2025_64988_MOESM1_ESM.pdf]

Supplemental Information for the manuscript: Childhood gut microbiome is linked to internalizing symptoms at school age via the functional connectome

Supplementary Methods

Participant and Study Design

The GUSTO study recruited pregnant citizens and permanent residents of Singapore during their antenatal dating ultrasound scan appointment in the first trimester of pregnancy at one of two major public maternity hospitals in Singapore between June 2009 and September 2010[1]. To be eligible to participate in the GUSTO study, pregnant women had to be at least 18 years of age, belong to one of the three main ethnic groups in Singapore (i.e., be of Chinese, Indian, or Malay ethnicity), be a Singaporean permanent resident or citizen, and intend to deliver their child at one of the two study recruitment hospitals. Because the original aim of GUSTO was to examine genetic factors contributing to differences in rates of type II diabetes across ethnic groups, infants of mixed ethnicity (i.e., the infant's mother and father were of different ethnicities) were not eligible to participate. Women with a Type I diabetes diagnosis, who were currently undergoing chemotherapy, or were taking psychotropic drugs were also excluded from participation.

Data relevant to this paper was collected in 2011-2013 (stool samples), 2015-2017 (fMRI scans), and 2017-2018 (caregiver-reported internalizing symptoms).

Measures

Caregiver-Reported Internalizing Symptoms: Child Behavior Checklist (CBCL)

Following scoring manual recommendations for the use of CBCL in research,[2] raw scores (as opposed to t-scores) were used. A box-cox transformation was applied to scores before analysis in order to reduce skew. The optimal transformation parameter (λ = .262) was estimated with maximum likelihood using the MASS R package.[3]

Caregiver-Reported Externalizing Symptoms: Child Behavior Checklist

Caregivers reported on their children's externalizing symptoms at age 7.5 years using the externalizing problems subscale of the CBCL (α = .85). The externalizing subscale is a composite of the social problems, thought problems, and attention problems subscales.

Caregiver-Reported Depressive Symptoms: Child Behavior Checklist

The 13-item CBCL DSM-oriented depressive problems subscale was used to assess caregiver-reported depressive symptoms at child age 7.5 years (α = .58).

Caregiver-Reported Anxiety Symptoms: Child Behavior Checklist

The 9-item CBCL DSM-oriented anxiety problems subscale was used to assess caregiver-reported anxiety symptoms at child age 7.5 years (α = .62).

Maternal Mental Health (child age 6 years)

Mothers reported on their own mental health when their children were age 6 years using the the State-Trait Anxiety Inventory (STAI[4]) and the Beck Depression Inventory 2 (BDI-2[5]). The 20-item state subscale of the STAI was used to assess anxiety symptoms, and the 21-tiem BDI-2 was used to assess depressive symptoms. Responses to all items on each scale were summed to create a total score, with higher scores indicating greater severity of anxiety and depressive symptoms, respectively.

Child Self-Reported Anxiety Symptoms (age 8.5 years)

Children reported on their anxiety symptoms at age 8.5 years using the Multidimensional Anxiety Scale for Children - Second Edition (MASC-2[6]). The MASC-2 has 50 items, each of which is rated on a 4-point Likert scale (0 = never, to 3 = often). Responses were summed to form a total score (α = .92).

Child Self-Reported Depressive Symptoms (age 8.5 years)

Children reported on their depressive symptoms at age 8.5 years using the Child Depression Inventory-2 (CDI-2[7]. The CDI-2 has 28 items, each of which is rated from 0 (no symptoms) to 2 (definite symptoms). Responses from all items were summed to create a total score (α = .81).

Monthly Income Per Household Member

Monthly income per household member was estimated as the median of the household income bin indicated by the responding parent ($0-$999, $1000-1999, $2000-3999, $4000-5999, or $6000 or more) divided by the number of people reported to live in the child's household.

Medication Use

Besides antibiotics and probiotics, other medications reported in this sample were limited to short-term use of oral medications (antihistamines, pain relievers, decongestants, cough suppressants), steroid creams, and inhalers for acute infections, allergies, eczema, and wheezing. See Table 1 for information on duration and number of medications reported.

Covariates

Variables that have previously been associated with composition of the gut microbiota in early life, childhood brain development or fMRI data quality, and/or child internalizing symptoms were included a priori as covariates in analyses: child gestational age at birth,[8,9] birthweight,[10] birth method (i.e., cesarean section or vaginal),[11,12] maternal education (see below),[13,14], and sex,[15] obtained from hospital birth records, child consumption of macronutrients (i.e., protein, fat, carbohydrates, and fiber) in the diet at 18 months of age,[16] and age that the child stopped breastfeeding (<1 month, 1-3 months, 3-6 months, 6-12 months, or >12 months,[12] exposure to antibiotics and probiotics at 2 years of age[17], and mean framewise displacement (FD) at the fMRI scan.[18] Because of the high number of potential diet-related covariates, macronutrients and age stopped breastfeeding were selected via a data-driven process, described below in the section "Covariate Selection for Diet Variables".

Because a small percentage (14.5%, 8 of 55) of participants had exposure to antibiotics or probiotics, instead of controlling for those variables we performed sensitivity analyses excluding participants with antibiotic or probiotic exposure. Results are reported below in Supplemental Results, "Sensitivity Analyses" section.

Maternal Education

Mothers selected their highest level of education attained from 6 categories: No education, Primary (PSLE), Secondary (GCE O/N Levels), ITE/NITEC, GCE A Levels/Polytechnic/Diploma, and University (Bachelors, Masters, PhD). For statistical analysis, a binary variable was created with 2 categories: less than a university education (68% of those who responded), and at least a university education (32% of those who responded).

Missing Data

Within the analytic sample, 22% (12 out of 55 participants) were missing data on the age at which breastfeeding was stopped, 22% (12 out of 55 participants) were missing data on child diet, and 9% (5 out of 55 participants) were missing data on maternal education. There was no missing data on child sex, gestational age at birth, birthweight, or birth method, and all included individuals had a microbiota sample, brain scan, and internalizing symptom report. Participants with missing data were dropped from analyses that included covariates (linear regression and mediation models).

MRI Data Acquisition

As previously described in Uy et al.,[18] MRI images were acquired at KK Women's and Children's Hospital and Clinical Imaging Research Centre at the National University of Singapore using a 3T Siemens Magnetom Skyra scanner with a 32-channel head coil. T1-weighted Magnetization Prepared Rapid Gradient Recalled Echo images (MPRAGE; 160 slices, 1 mm thickness, field-of-view = 192 × 192 mm2 , matrix = 192 × 192, repetition time = 2000 ms, echo time = 2.08 ms, inversion time = 877 ms, flip angle = 9◦, scanning time = 3.5 min) and resting state (rs) fMRI images (single-shot echo-planar imaging; 48 slices with 3 mm slice thickness, no interslice gaps, matrix = 64 × 64, field- of-view = 192×192 mm2, echo time = 27 ms, flip angle = 90◦, repetition time = 2.62–2.66 sec, scanning time = 5.27 min) were acquired. Children were asked to close their eyes during the rs-fMRI scan.

Stool Sample Collection

Children donated stool samples at age 2 years. Caregivers were provided with collection kits and instructed on how to use the kits at an in-person study visit. Fresh stool samples were collected by each child's caregiver in their home using sterile collection containers and then refrigerated at 4℃ before being cold-chain transported to the lab < 24 hours after collection. Once in the lab, they were stored in a -80 ℃ freezer until further processing.[19,20] No preservatives or buffers were added for storage.

Gut Microbiota DNA Extraction and Sequencing

Detailed descriptions of DNA extraction and sequencing performed in the GUSTO study on 2 year stool samples can be found in Chen el al.[21] Briefly, DNA extraction was performed at the University of Chicago Microbiome Center with MoBio PowerFecal DNA kits.[22] The V4 region of the 16S rRNA gene (a marker of bacterial DNA) was amplified with polymerase chain reaction using modified 515F and 806R primers. Amplicon sequencing was then performed in two batches using the Illumina MiSeq platform standard protocol[23] to generate 250 bp paired-end reads. Sequence data were de-multiplexed to determine the sample origin of each read and reads were clustered into operational taxonomic units (OTUs) using USEARCH v9.2.64 at a 97% similarity threshold. No controls or replicates were used. No methods were used to control for or identify contamination, but all samples had sufficient microbial load, making contamination less relevant. A taxonomic classification was assigned to each OTU by comparing against the SILVA 123 ribosomal reference database (https://www.arb-silva.de/). There were no differences in any study variables of interest across the two sequencing batches.

MRI Data Preprocessing

We used FSL version 5.0.9 to preprocess MRI data. As described in Uy et al.,[18] rs-fMRI preprocessing steps involved skull stripping, slice time and motion correction, and intensity normalization.[24] We discarded the first four volumes to allow magnetization to reach equilibrium. We used fsl_motion_outliers to generate confound matrices to censor volumes that exceeded 0.2 mm framewise displacement (FD), which we included in the general linear model. We excluded scans that had greater than 36 (30%) frames exceeding 0.2 mm FD and/or any frames exceeding 2mm FD (N = 53 of N = 110 collected scans were excluded).[25] We then visually inspected all rs-fMRI scans to ensure that the brain was sufficiently in view and there were no large-scale motion artifacts (no scans were excluded in this stage). Finally, for remaining subjects, we visually inspected each subjects' structural scan for the presence of motion artifacts and excluded scans with extensive artifacts (N = 2 additional scans excluded).

We used FSL's Automatic Segmentation Tool (FAST[26]) to segment the structural image into gray matter, white matter, and cerebrospinal fluid masks. After transforming masks into each subjects' functional space, we extracted signals from those masks and included them as nuisance regressors along with their temporal derivatives to account for the global signal[25]. We also included standard and extended motion parameters (24 parameters) as nuisance regressors. For functional images, preprocessing included temporal high-pass filtering using a 0.01 Hz filter, smoothing using a 6 mm full-width half maximum Gaussian kernel, and linear registration to the structural image (with 6 degrees of freedom).

Microbiota Functional Prediction

Metagenomic functional prediction was performed using PICRUSt v2.6.2[27]. Two OTUs were removed during PiCRUST: one OTU that had poor alignment to reference sequences, and one OTU that was above the Nearest Sequenced Taxon Index (NSTI) cutoff for similarity to reference genomes of 2.0. The average weighted NSTI score for analyzed samples was 0.012, meaning that the average microbe in the sample could be predicted using a relative from the same (98.98%) species and suggesting the predictions were high quality. Predicted MetaCyc pathways, regrouped from Enzyme Commission numbers, were used for downstream analysis. Prior to downstream analysis, rare pathways where the sum of abundances were below 5% of the total abundances in the dataset were removed (keeping 364 out of 504 total pathways) and counts for each pathway were center-log-ratio transformed to bring the data into unbounded space.

Data Analysis Plan

Covariate Selection for Diet Variables

The data-driven selection process for diet covariates included two steps. First, we narrowed down the list of potential diet covariates to those that were significantly associated with either caregiver-reported internalizing symptoms, brain-internalizing signatures, and/or gut microbiota alpha diversity according to bivariate correlations in the case of continuous variables or t-test or ANOVA in the case of categorical variables. We used alpha diversity as the microbiota variable for diet covariate selection because this microbiota feature was not derived from the brain or internalizing symptoms (in contrast to the microbial profile scores). Variables selected in step 1 were: consumption of polyunsaturated fat, carbohydrates, total fat, and fiber.

Second, we flagged any pair of variables selected in step 1 that were highly correlated ($r > |.8|$). For pairs of variables flagged in step 2, one variable of the two was selected based on either maximal sample size or strength of association with the primary study variables. Consumption of carbohydrates and total fat were flagged because they were highly correlated ($r = -.86$) and total fat had a stronger association with the SOFA Inter-Network brain signature than did carbohydrates ($r = -.36$ vs. .30), so total fat was selected. Thus, the final set of selected diet covariates included consumption of fiber, total fat, and polyunsaturated fat.

Correlations between primary study variables and continuous diet covariates can be found in Figure S1, and bivariate associations (*t*-tests and ANOVAs) of age stopped breastfeeding with primary study variables can be found in Table S1. All tests were 2-tailed with a significance threshold of $p < .05$.

Outlier Identification

Scatterplots of scores for each derived component in final sPLS models vs. the outcome variable were visually examined for multivariate outliers. Any multivariate outliers were tested for univariate outlier values on any variable in the predictor dataset, and were winsorized to the next highest value in the sample on variables for which they were univariate outliers. Values above the third quartile + 1.5* the interquartile range or below the first quartile - 1.5* the interquartile range for the sample were classified as outliers. Model tuning was then repeated on the winsorized dataset.

Demographic characteristics and other variables of interest (e.g., caregiver-reported internalizing symptoms) were examined for the participants identified as outliers to determine whether those participants were outliers (continuous variables) or members of uncommon categories (categorical variables) on any other characteristics.

Differences Between Included and Excluded Samples

T-tests for continuous variables and chi-square tests for categorical variables were used to evaluate bivariate differences in variables of interest and covariates for the analytic sample compared to the sample of children that participated in the year 6 fMRI scan, but whose data were excluded in fMRI quality control (Table 1). The only significant difference between included and excluded children was that excluded children had higher average mean FD, which is expected given that a mean FD cutoff was used as an exclusion criterion in the fMRI quality control (see “MRI Data Preprocessing”).

Regression Models

A standardized coefficient (β) representing the strength and direction of association of each sPLS component with its corresponding outcome variable controlling for covariates, is reported, as well as the standard error (SE) and p-value of that coefficient. β values between 0.10 and 0.29 are considered small, values between 0.30 and 0.49 are considered medium, and values greater than or equal to 0.50 are considered large.[28]

Brain Signature and Microbial Profile Associations with other Mental Health Domains and Reporters

Bivariate correlations were computed evaluating associations between each derived brain signature and each microbial profile with other caregiver-reported mental health domains at age 7.5 years: externalizing symptoms, depressive symptoms, and anxiety symptoms, as well as with other reporters at age 8.5 years: child self-reported depressive symptoms and anxiety symptoms. For any significant associations, multiple linear regression models were fitted predicting the symptom variable from the brain or microbiota variable controlling for covariates, to test whether associations were robust to inclusion of covariates.

Calculation of Microbial Profile Scores in Sample with Microbiota and Caregiver-Reported Internalizing Symptom Data

We used the predict() function from the MixOmics R package to generate Microbial Profile scores for participants from the GUSTO study who provided a usable stool sample at age 2 years, and a caregiver-reported internalizing symptoms assessment at age 7.5 years (N=318) using the sPLS models for the Microbial Profiles derived in the main results section.

Supplementary Notes

Caregiver-Reported Internalizing Symptom Associations with Child Self Reported Symptoms at age 8.5 years

Caregiver-reported internalizing symptoms at age 7.5 were significantly positively correlated with child self-reported depressive symptoms at age 8.5 years (r = .41, $t(31)$ = 2.47, p = .02) and marginally correlated with child self-reported anxiety symptoms at age 8.5 years (r = 0.30, $t(30)$ = 1.72, p = .095).

Characteristics of Participants Flagged as Outliers on Brain Networks or Microbiota Genera

The two participants identified as outliers on several rsFC network values were not outliers on any other continuous variables of interest or demographic characteristics. However, they did belong to some of the more uncommon demographic categories - one of the two outlier participants took antibiotics at age 2 years (13% of sample) and the other took probiotics at age 2 years (5% of sample).

The participant identified as an outlier on abundance of several microbiome genera was also a high outlier on one of the alpha diversity metrics - observed features. Otherwise, this child was not an outlier on any other continuous variables of interest or demographic characteristics, and was not a member of any uncommon demographic categories.

Brain Signature Associations with Other Mental Health Domains and Reporters

Scores on the SOFA, MTL, SAL, PMN Network Connectivity Brain Signature were positively associated with caregiver-reported externalizing symptoms (r=.39, $t(53)$ = 3.06, p=.003), depressive symptoms (r=.36, $t(53)$ = 2.81, p<.001), and anxiety symptoms (r=.38, $t(53)$ = 2.98, p=.005). They were also positively associated with child self-reported depressive symptoms (r=.47, $t(30)$ = 2.91, p=.005), but not anxiety symptoms (r=-.22, $t(31)$ = -1.23, p=.18), at age 8.5 years. The association with caregiver-reported anxiety symptoms was robust to inclusion of covariates (β = 0.42, SE = 0.19, p = .031), but the other associations were not:

caregiver-reported externalizing symptoms (β = 0.44, SE = 0.23, p = .060), depressive symptoms (β = 0.45, SE = 0.28, p = .12), and child self-reported depressive symptoms (β = 0.75, SE = 0.36, p = .061).

Scores on the SOFA Between Network Connectivity Brain Signature were not associated with caregiver-reported externalizing symptoms (r=.29, $t(53)$ = 2.21, p=.096), depressive symptoms (r=.10, $t(53)$ = 0.74, p=.47), or anxiety symptoms (r=.22, $t(53)$ = 1.67, p=.20). However, they were positively associated with child self-reported depressive symptoms (r=.32, $t(30)$ = 1.87, p=.02), but not anxiety symptoms (r=.08, $t(31)$ = 0.43, p=.56), at age 8.5 years. The association with child self-reported depressive symptoms was not robust to the inclusion of covariates (β = 0.31 SE = 0.51, p = .56).

Microbial Profile Associations with Other Mental Health Domains and Reporters

Scores on Microbial Profile 1 were positively associated with caregiver-reported externalizing symptoms (r = .20, $t(53)$ = 1.81, p= .033), but not anxiety symptoms (r = .06, $t(53)$ = 0.47, p= .66) or depressive (r = .09, $t(53)$ = 0.64, p= .44) symptoms. They were also not associated with self-reported depressive (r = -.22, $t(30)$ = -1.25, p= .29) or anxiety (r = .02, $t(31)$ = 0.091, p= .94) symptoms at age 8.5 years. The positive association with caregiver-reported externalizing symptoms was not robust to inclusion of covariates (β = 0.20, SE = 0.18, p = .26).

Scores on Microbial Profile 2 were not associated with caregiver-reported externalizing symptoms (r = .10, $t(53)$ = 0.76, p= .51), anxiety symptoms (r = .14, $t(53)$ = 1.05, p= .35), or depressive symptoms (r = .05, $t(53)$ = 0.39, p= .65); or self-reported depressive symptoms (r = .04, $t(30)$ = 0.21, p= .84) at a later time point (age 8.5 years). Scores were marginally positively associated with self-reported anxiety symptoms (r = .29, $t(31)$ = 1.70, p=.080) at age 8.5 years.

Scores on Microbial Profile 3 were marginally positively associated with caregiver-reported externalizing symptoms (r =.21, $t(53)$ = 1.58, p= .090). Scores were not associated with caregiver-reported anxiety symptoms (r = .11, $t(53)$ = 0.78, p= .45) or depressive symptoms (r = .11, $t(53)$ = 0.80, p= .33); or self-reported depressive symptoms (r = .14, $t(30)$ = 0.77, p= .45), or self-reported anxiety symptoms (r = -.37, $t(31)$ = 1.51, p= .18) at age 8.5 years.

Microbial Profile Associations with Caregiver-Reported Internalizing Symptoms in Larger Sample

In the larger GUSTO study sample of participants who donated a usable stool sample at age 2 years and whose caregiver provided an internalizing symptom assessment at age 7.5 years (N=318), we tested the association between scores on each microbial profile and caregiver-reported internalizing symptoms. As in the analytic sample, there were no significant overall associations between any microbial profile and caregiver-reported internalizing symptoms, controlling for covariates: Microbial Profile 1 (β= -0.01, SE= 0.13, p=.93), Microbial Profile 2 (β= 0.13, SE=0.11, p=.22), or Microbial Profile 3 (β=-0.24, SE=0.14, p=.30).

Predicted Microbiota Functional Profiles That Maximally Covaried with Brain Signatures

Predicted microbial functional pathways associated with SOFA, MTL, SAL Intra-Network Connectivity Brain Signature: There were no multivariate outliers. The final best-fitting model consisted of 1 microbiota functional component with 5 pathways. This component was

correlated with the SOFA, MTL, SAL, PMN Network Connectivity Brain Signature (*r* =.34, *t*(53) = 2.64, *p* = .011) and was significantly positively related to its corresponding brain signature when controlling for covariates (β=0.48, SE = 0.17, p = .024). This component consisted of 5 predicted functional pathways, all with negative loadings. One of the pathways had high stability: PWY-6270, isoprene biosynthesis I. We named this component “Functional Profile 1”.

Predicted microbial functional pathways associated with SOFA Inter-Network Connectivity Brain Signature: There were no multivariate outliers. The final best-fitting model consisted of 2 microbiota functional components. Each component included 5 pathways, 3 of which had high stability.

Component 1 was correlated with the SOFA Between Network Connectivity Brain Signature (*r* = .41, *t*(53) = 3.24, *p* = .002) and was not significantly related to its corresponding brain signature when controlling for covariates (β=0.13, SE = 0.18, p = .47). The top 3 highest magnitude loadings for this component, which had high stability, consisted of positive loadings for PWY-7377:cob(II)yrinate a,c-diamide biosynthesis I (early cobalt insertion), and negative loadings for PENTOSE-P-PWY: pentose phosphate pathway and PWY-6901: superpathway of glucose and xylose degradation. We named this component “Functional Profile 2”.

Component 2 was correlated with the SOFA Between Network Connectivity Brain Signature (*r* = .47, *t*(53) = 3.43, *p* = .0012) and was significantly positively related to its corresponding brain signature when controlling for covariates (β=0.32, SE = 0.17, p = .037). The top 3 highest magnitude loadings for this component, which had high stability, consisted of positive loadings for PWY3O-4107: NAD salvage pathway V, PYRIDNUCSAL-PWY: NAD salvage pathway I, and UDPNAGSYN-PWY: UDP-N-acetyl-D-glucosamine biosynthesis I. We named this component “Functional Profile 3”.

See Table S4 for loadings, VIP, and stability values for all pathways loading onto each functional profile.

There were no significant total, direct, or indirect associations (via the corresponding brain signature) between the functional profiles and caregiver-reported internalizing symptoms at age 7.5 years. Full mediation results are included in Table S6.

Sensitivity Analyses: Excluding Participants who Took Antibiotics or Probiotics at age 2 years

Because the two participants who were outliers on brain networks were reported to have taken antibiotics or probiotics at age 2 years (the time point concurrent to the microbiota sample), and such medication could have disrupted the microbiota in ways that might affect the results of this study, we repeated the main study multiple regression analyses excluding participants (N=8) who were reported to have taken antibiotics and/or probiotics at age 2 years. These sensitivity analyses assessed whether relationships between microbial profiles, brain signatures, and caregiver-reported internalizing symptoms held in the subsample of children without reported exposure to microbiota-altering medications at age 2 years.

In the subsample of participants without antibiotic probiotic exposure at age 2 years, the SOFA, MTL, SAL, PMN Network Connectivity Brain Signature remained significantly positively related to caregiver-reported internalizing symptoms, controlling for covariates (β=0.42, SE=0.22, *p*=.047). The SOFA Between Network Connectivity Brain Signature also remained significantly positively related to caregiver-reported internalizing symptoms, controlling for

covariates (β=0.60, SE=0.20, p=.006). Microbial Profile 1 remained significantly positively related to its corresponding brain signature, the SOFA, MTL, SAL, PMN Network Connectivity Brain Signature, controlling for covariates (β=0.46, SE=0.25, *p*=.047). Likewise, Microbial Profiles 2 (β=0.65, SE=0.23, *p*=.009) and 3 (β=0.47, SE=0.19, *p*=.020) remained significantly positively related to the SOFA Between Network Connectivity Brain Signature controlling for covariates.

In the subsample of participants without antibiotic exposure at age 2 years, associations between the Microbial Profiles and caregiver-reported internalizing symptoms remained very similar to results in the full analytic sample, except that Microbial Profile 2 was significantly associated with caregiver-reported internalizing symptoms controlling for covariates (β=0.66, SE=0.23, *p*=.008), though there was no significant association in the full analytic sample. Microbial Profiles 1 (β=0.34, SE=0.22, *p*=.14) and 3 (β=0.21, SE=0.30, *p*=.50) remained not significantly associated with caregiver-reported internalizing symptoms controlling for covariates.

Comparison of Effect Sizes from Mediation Models to Prior Research

We compared partial R^2 values from each path in the mediation models (Table S5) to other studies to assess whether the amount of variance explained in each path is reasonable. Partial R^2 for the *a* paths (gut microbiome to brain) in our study ranged from 0.013 to 0.29, which is similar to values in other studies on the gut microbiome and neuroimaging in childhood that reported partial R^2. [8,29] Partial R^2 for the *b* paths (brain to internalizing symptoms) ranged from 0.06-0.22, which is similar to or higher magnitude than the other study that reported on rsFC and internalizing symptom associations in childhood.[30] The higher values we found could be because Albertina et al. examined individual pairs of ROIs within- and between-networks, whereas our analysis approach (i.e., generating brain signatures using sPLS) incorporates information from multiple ROI pairs in the same model. Based on this comparison, we believe that our models are adequately capturing the patterns in the data.

Supplementary Figures

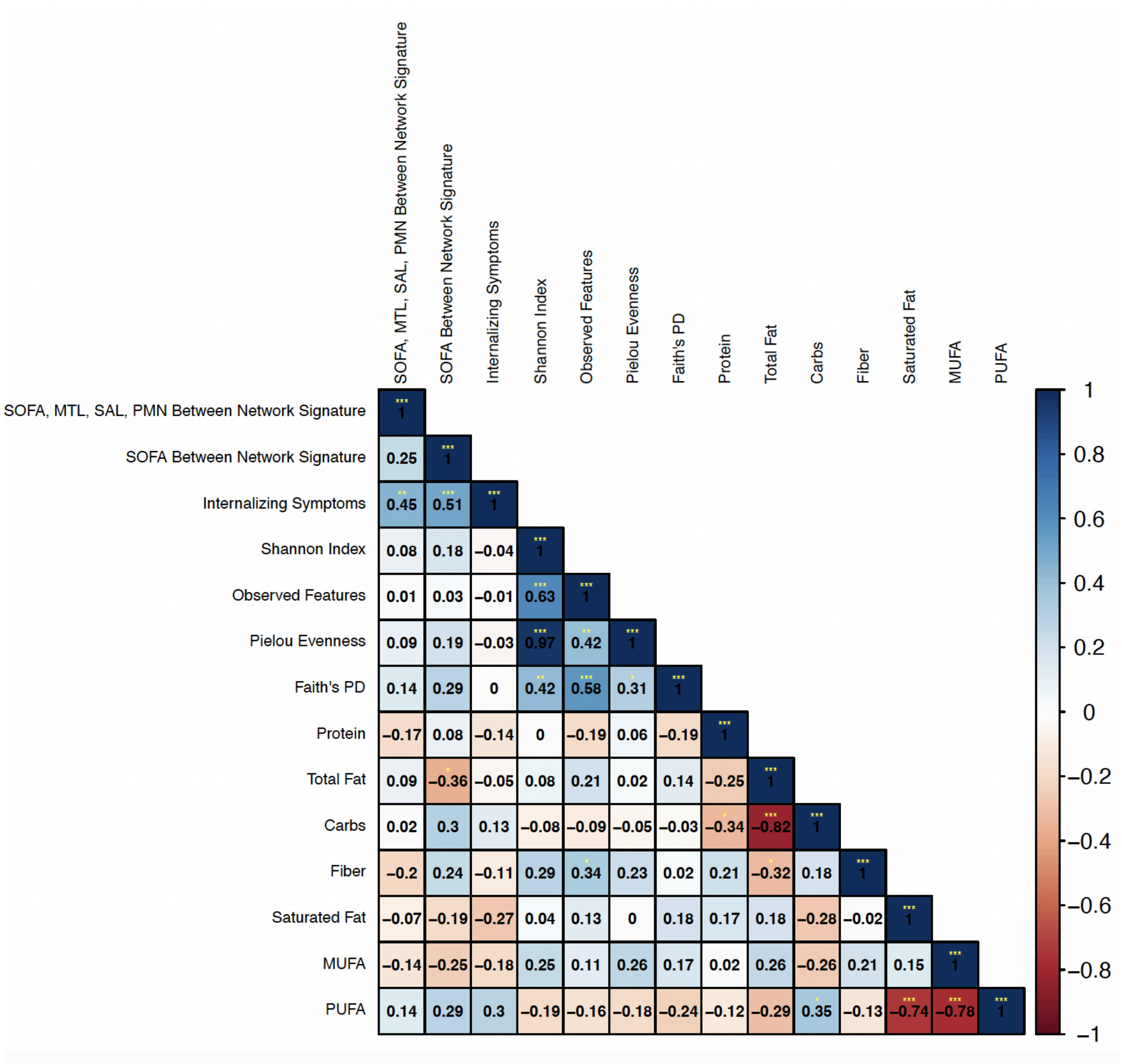

Supplementary Fig. 1. **Bivariate correlations between potential continuous diet covariates and primary study variables.** Correlations are Pearson's *r* values. * indicates $p < .05$, ** indicates $p < .01$, ***indicates $p < .001$. Faith's PD = Faith's phylogenetic diversity, PUFA = polyunsaturated fat, MUFA = monounsaturated fat. N = 55 samples.

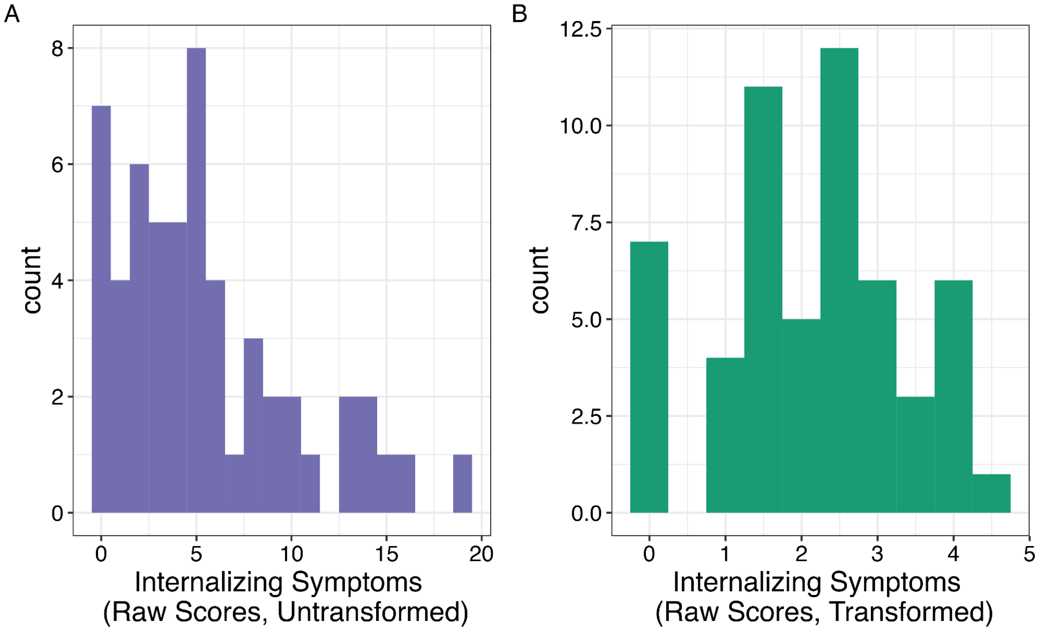

Supplementary Fig. 2. **Distribution of caregiver-reported internalizing symptom raw scores**. **A** scores before (purple) Box-Cox transformation. **B** scores after (green) Box-Cox transformation. N=55 samples. Source data are provided as a Source Data file.

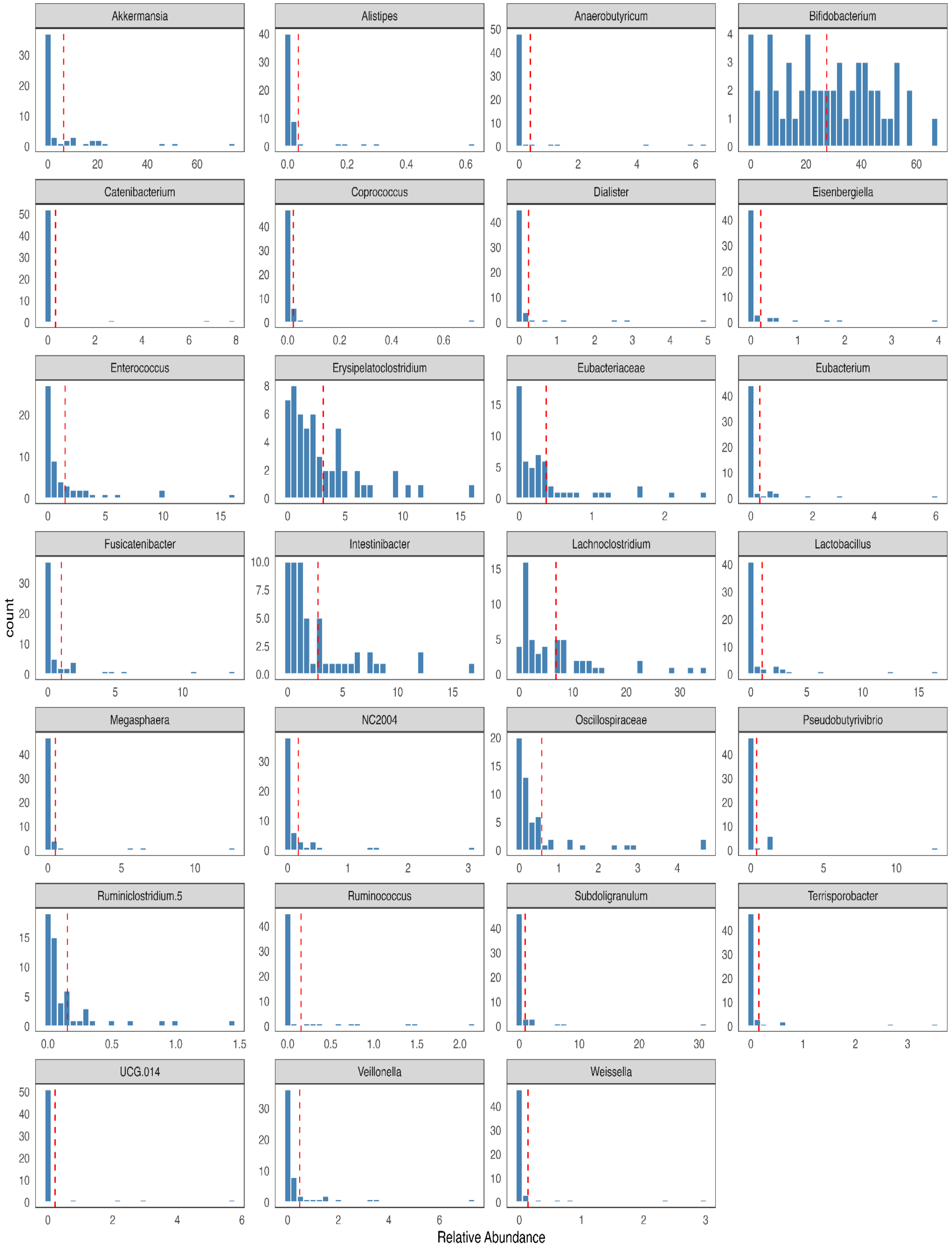

Supplementary Fig. 3. **Histograms showing distribution of relative abundance for each genus selected for inclusion in the Microbial Profiles.** Dotted red lines indicate average

relative abundance value in the sample. N = 55 samples. Source data are provided as a Source Data file.

Supplementary Tables

Supplementary Table 1. **ANOVAs testing associations of age stopped breastfeeding with primary study variables.**

Variable	*F* statistic	*p* value
SOFA, MTL, SAL, PMN Network Connectivity Brain Signature	1.86	.14
SOFA Between Network Connectivity Brain Signature	0.28	.89
Caregiver-Reported Internalizing Symptoms	0.53	.73
Shannon Index	1.25	.31
Observed Features	1.11	.37
Pielou Evenness	1.10	.38
Faith's PD	1.58	.20

Note. ANOVA tests use a one-tailed *F*-distribution.

Supplementary Table 2. **Loadings and VIPs for each variable onto each brain signature**

Network	Loading	VIP	Stability
SOFA, MTL, SAL, PMN Network Connectivity Brain Signature			
SOFA	0.52	5.0	1
SAL_PMN	-0.52	5.0	1
SOFA_MTL	0.37	3.50	1
SAL	-0.3	2.80	1
MTL	0.24	2.30	0.8
SAL_SMD	-0.21	2.20	1
CON_PMN	-0.2	1.90	1
SAL_AUD	-0.17	1.60	0.9
VAN_PMN	-0.15	1.50	0.9
DMN_SAL	-0.12	1.10	0.9
SAL_SML	-0.076	0.72	0.8

SAL_VAN	-0.06	0.57	0.8
MTL_PMN	-0.053	0.51	0.6
DMN_MTL	0.053	0.50	0.7
CON_SMD	-0.047	0.45	0.7
DMN_SOFA	0.034	0.33	0.7
SAL_DAN	-0.012	0.12	0.6
DAN_VIS	-0.0089	0.084	0.5
VIS_SMD	-0.0059	0.057	0.6
SAL_CON	-0.0026	0.025	0.4
SOFA Between Network Connectivity Brain Signature			
VIS_SOFA	0.55	3.00	0.8
DMN_SOFA	0.51	2.80	0.8
VAN_SMD	0.36	2.00	0.8
FPN_SOFA	0.27	1.50	0.8
VAN_SOFA	0.25	1.40	0.8
CON_SOFA	0.2	1.10	0.8
SMD_SML	0.19	1.00	0.8
DAN_SML	0.18	1.00	0.8
VAN_MTL	0.15	0.83	0.8
DAN_VAN	0.12	0.65	0.5
SOFA_PMN	0.11	0.63	0.5
DMN	0.068	0.37	0.6
VIS_PMN	0.068	0.37	0.4
DAN_SOFA	0.064	0.36	0.4
DMN_CON	0.057	0.32	0.6

Supplementary Table 3. **Loadings, VIPs, stability values and N not zero for each variable selected in the microbial profiles**

Class	Order	Family	Genus	Loading	VIP	Stability	N Non Zero Counts
Microbial Profile 1							
Clostridia	Clostridiales	Eubacteriaceae	Unknown	0.73	5.89	1.0	42
Clostridia	Clostridiales	Eubacteriaceae	Anaerobutyricum	-0.59	4.74	1.0	43
Bacilli	Lactobacillales	Leuconostocaceae	Weissella	-0.30	2.43	0.9	22
Clostridia	Clostridiales	Lachnospiraceae	Coprococcus	-0.11	0.86	0.6	11
Negativicutes	Selenomonadales	Veillonellaceae	Dialister	-0.053	0.43	0.4	26
Microbial Profile 2							
Clostridia	Clostridiales	Ruminococcaceae	Eubacterium	0.93	7.41	1.0	24
Clostridia	Clostridiales	Peptostreptococcaceae	Terrisporobacter	0.37	2.94	0.9	16
Bacilli	Lactobacillales	Lactobacillaceae	Lactobacillus	-0.061	0.49	0.4	38
Negativicutes	Selenomonadales	Veillonellaceae	Dialister	0.050	0.40	0.7	26
Bacilli	Lactobacillales	Enterococcaceae	Enterococcus	-0.0079	0.063	0.2	55
Microbial Profile 3							
Erysipelotrichia	Erysipelotrichales	Erysipelotrichaceae	Catenibacterium	-0.4	2.2	1	17
Negativicutes	Selenomonadales	Veillonellaceae	Veillonella	0.36	2	1	48
Clostridia	Clostridiales	Lachnospiraceae	Fusicatenibacter	-0.36	1.9	1	44
Clostridia	Clostridiales	Peptostreptococcaceae	Intestinibacter	0.33	1.8	1	55
Clostridia	Clostridiales	Lachnospiraceae	Eisenbergiella	0.31	1.7	0.9	36
Clostridia	Clostridiales	Lachnospiraceae	Lachnoclostridium	0.3	1.6	1	55

Clostridia	Clostridiales	Lachnospiraceae	Coprococcus	0.25	1.3	0.9	11
Clostridia	Clostridiales	Ruminococcaceae	Ruminiclostridium.5	0.24	1.3	0.9	43
Clostridia	Clostridiales	Lachnospiraceae	Pseudobutyrivibrio	-0.24	1.3	0.9	27
Clostridia	Clostridiales	Lachnospiraceae	Ruminococcus	-0.2	1.1	1	33
Clostridia	Eubacteriales	Oscillospiraceae	Subdoligranulum	-0.15	0.83	0.9	18
Bacilli	Erysipelotrichales	Erysipelatoclostridiaceae	Erysipelatoclostridium	0.13	0.71	0.9	55
Clostridia	Clostridiales		UCG.014	-0.11	0.59	0.9	14
Actinomycetes	Bifidobacteriales	Bifidobacteriaceae	Bifidobacterium	-0.092	0.5	0.7	55
Negativicutes	Selenomonadales	Veillonellaceae	Dialister	0.063	0.45	0.5	26
Clostridia	Eubacteriales	Oscillospiraceae	Unknown	0.045	0.25	0.7	52
Bacteroidia	Bacteroidales	Rikenellaceae	Alistipes	-0.044	0.24	0.8	15
Clostridia	Clostridiales	Lachnospiraceae	NC2004	-0.037	0.2	0.6	21
Verrucomicrobiae	Verrucomicrobiales	Akkermansiaceae	Akkermansia	-0.027	0.15	0.3	51
Negativicutes	Selenomonadales	Veillonellaceae	Megasphaera	-0.016	0.087	0.5	23

Note. Average abundances are rounded to two significant figures. Cells in color highlight higher-level taxonomic trends: many microbes are from the Clostridiales order (blue highlight), and the Lachnospiraceae (red highlight) or Ruminococcaceae (yellow highlight) families within Clostridiales.

Supplementary Table 4. **Loadings, and VIPs, and stability values for variables selected in the predicted functional profiles**

MetaCyc Pathway	Pathway Description	Loading	VIP	Stability
Functional Profile 1				
PWY-6270	isoprene biosynthesis I	-0.70	10.87	0.8
PYRIDNUCSAL-PWY	NAD salvage pathway I	-0.44	6.78	0.6
PWY-5265	peptidoglycan biosynthesis II (staphylococci)	-0.38	5.93	0.6
PWY-7392	taxadiene biosynthesis (engineered)	-0.38	5.92	0.7
HEXITOLDEGSUPER-PWY	superpathway of hexitol degradation (bacteria)	-0.12	1.90	0.2
Functional Profile 2				
PENTOSE-P-PWY	pentose phosphate pathway	-0.68	10.51	1
PWY-6901	superpathway of glucose and xylose degradation	-0.50	7.71	0.9
PWY-7377	cob(II)yrinate a,c-diamide biosynthesis I (early cobalt insertion)	0.40	6.20	0.9
PWY-7234	inosine-5'-phosphate biosynthesis III	-0.33	5.05	0.7
PWY-8011	L-serine biosynthesis II	0.13	2.01	0.2
Functional Profile 3				
PWY3O-4107	NAD salvage pathway V	0.81	9.01	1
PYRIDNUCSAL-PWY	NAD salvage pathway I	0.49	5.42	0.9
UDPNAGSYN-PWY	UDP-N-acetyl-D-glucosamine biosynthesis I	0.30	3.37	0.8
PWY-5345	superpathway of L-methionine biosynthesis (by sulfhydrylation)	-0.14	1.59	0.5
P4-PWY	superpathway of L-lysine, L-threonine and L-methionine biosynthesis I	0.030	0.33	0.4

Supplementary Table 5. **Coefficient estimates from mediation models testing effects of microbial profiles and alpha diversity on caregiver-reported internalizing symptoms via corresponding brain signatures**

Model: Microbial Profile 1 → SOFA, MTL, SAL, PMN Network Connectivity Brain Signature → Caregiver-Reported Internalizing symptoms

	b	**β**	**Standard Error**	***p* or Standardized 95% CI**	**Partial R^2**
a path	0.50	0.46	0.21	.013	0.13
b path	0.33	0.45	0.14	.031	0.17
Indirect effect	0.16	0.16	0.13	[-0.061, 0.43]	
Direct effect	0.18	0.19	0.20	[-0.22, 0.59]	
Total effect	0.28	0.46	0.24	.065	0.070

Model: Microbial Profile 2 → SOFA Between Network Connectivity Brain Signature → Caregiver-Reported Internalizing Symptoms

	b	**β**	**Standard Error**	***p* or Standardized 95% CI**	**Partial R^2**
a path	0.83	0.48	0.21	.032	0.29
b path	0.20	0.35	0.23	.23	0.062
Indirect effect	0.17	0.17	0.19	[-0.11, 0.65]	
Direct effect	0.29	0.29	0.24	[-0.20, 0.79]	
Total effect	0.47	0.46	0.24	.065	0.16

Model: Microbial Profile 3 → SOFA Between Network Connectivity Brain Signature → Caregiver-Reported Internalizing Symptoms

	b	**β**	**Standard Error**	***p* or Standardized 95% CI**	**Partial R^2**
a path	0.65	0.47	0.19	.020	0.23
b path	0.40	0.68	0.15	0.014	0.21
Indirect effect	0.26	0.32	0.17	[0.0075, 0.68]	
Direct effect	-0.24	-0.23	0.20	[-0.65, 0.16]	
Total effect	0.01	0.01	0.25	.96	0.0001

Model: Faith's Phylogenetic Diversity → SOFA, MTL, SAL, PMN Network Connectivity Brain Signature → Caregiver-Reported Internalizing symptoms

	b	**β**	**Standard**	***p* or**	**Partial R^2**

			Error	Standardized 95% CI	
a path	0.09	0.11	0.22	0.64	0.013
b path	0.37	0.50	0.13	0.012	0.22
Indirect effect	0.034	0.052	0.11	[-0.13, 0.32]	
Direct effect	0.10	0.10	0.11	[-0.12, 0.33]	
Total effect	0.13	0.21	0.20	0.31	0.044

Model: Faith's Phylogenetic Diversity → SOFA Between Network Connectivity Brain Signature → Caregiver-Reported Internalizing symptoms

	b	**β**	**Standard Error**	***p* or Standardized 95% CI**	**Partial R^2**
a path	0.41	0.37	0.12	.008	0.22
b path	0.30	0.51	0.13	.030	0.13
Indirect effect	0.12	0.19	0.14	[-0.084, 0.48]	
Direct effect	0.013	0.013	0.13	[-0.26, 0.28]	
Total effect	0.13	0.21	0.20	0.31	0.044

Note. Statistical tests were two-sided; no adjustment was made for multiple comparisons.

Supplementary Table 6. **Coefficient estimates from mediation models testing effects of predicted functional profiles on caregiver-reported internalizing symptoms via corresponding brain signatures**

Model: Functional Profile 1 → SOFA, MTL, SAL, PMN Network Connectivity Brain Signature → Caregiver-Reported Internalizing symptoms

	b	**β**	**Standard Error**	***p* or Standardized 95% CI**	**Partial R^2**
a path	0.41	0.48	.17	.024	.21
b path	0.29	0.40	.15	.062	.13
Indirect effect	0.19	0.15	.19	[-0.14, 0.67]	
Direct effect	0.21	0.21	.21	[-0.12, 0.54]	
Total effect	0.35	0.47	0.32	.16	.17

Model: Functional Profile 2 → SOFA Between Network Connectivity Brain Signature → Caregiver-Reported Internalizing Symptoms

	b	**β**	**Standard Error**	***p* or Standardized 95% CI**	**Partial R^2**
a path	0.18	0.13	0.18	.46	.21
b path	0.28	0.49	0.13	.042	0.15
Indirect effect	0.050	0.064	0.098	[-0.080, 0.30]	
Direct effect	0.18	0.18	0.15	[-0.13, 0.50]	
Total effect	0.23	0.30	0.25	.16	.072

Model: Functional Profile 3 → SOFA Between Network Connectivity Brain Signature → Caregiver-Reported Internalizing Symptoms

	b	**β**	**Standard Error**	***p* or Standardized 95% CI**	**Partial R^2**
a path	0.58	0.32	0.17	.037	.24
b path	0.30	0.51	0.14	.049	0.14
Indirect effect	0.19	0.16	0.14	[-0.11, 0.45]	
Direct effect	0.050	0.050	0.26	[-0.48, 0.57]	
Total effect	0.24	0.21	0.21	.34	.031

Note. Statistical tests were two-sided; no adjustment was made for multiple comparisons.

Supplementary References

1. Soh, S. E. *et al.* Cohort profile: Growing up in Singapore towards healthy outcomes (GUSTO) birth cohort study. *Int. J. Epidemiol.* **43**, 1401–1409 (2014).
2. Achenbach, T. M. & Rescorla, L. A. *Manual for Child Behavior Checklist for Ages 6-18*. (APA PsycTests, Burlington, VT, 2001).
3. W. N. Venables & B. D. Ripley. *Modern Applied Statistics with S-PLUS*. (Sporinger, 2002).
4. Spielberger, C. D., Gorsuch, R. L., Lushene, R., Vagg, P. R., & Jacobs, G. A. *Manual for the State-Trait Anxiety Inventory*. (Consulting Psychologists Press, Palo Alto, CA, 1983).
5. Beck, A. T., Ward, C. H., Mendelson, M., Mock, J. & Erbauch, J. Beck Depression Inventory. https://doi.org/10.1037/t00741-000 (2011).
6. March, J. S., Parker, J. D., Sullivan, K., Stallings, P. & Conners, C. K. The Multidimensional Anxiety Scale for Children (MASC): factor structure, reliability, and validity. *J. Am. Acad. Child Adolesc. Psychiatry* **36**, 554–565 (1997).
7. Kovacs, M. Childen's Depression Inventory 2nd Edition™. https://doi.org/10.1037/t04948-000 (2012).
8. Kelsey, C. M. *et al.* Gut microbiota composition is associated with newborn functional brain connectivity and behavioral temperament. *Brain. Behav. Immun.* **91**, 472–486 (2021).
9. Syn, N. L. *et al.* Severity of nausea and vomiting in pregnancy and early childhood neurobehavioural outcomes: The Growing Up in Singapore Towards Healthy Outcomes study. *Paediatr. Perinat. Epidemiol.* **35**, 98–108 (2021).
10. Sripada, K. *et al.* Trajectories of brain development in school-age children born preterm with very low birth weight. *Sci. Rep.* **8**, 15553 (2018).
11. Li, X. B. *et al.* Childhood trauma associates with clinical features of bipolar disorder in a sample of Chinese patients. *J. Affect. Disord.* **168**, 58–63 (2014).
12. Stewart, C. J. *et al.* Temporal development of the gut microbiome in early childhood from the

TEDDY study. *Nature* **562**, 583–588 (2018).

13. Lewis, C. R. *et al.* Family SES Is Associated with the Gut Microbiome in Infants and Children. *Microorganisms* **9**, 1608 (2021).
14. Holstein, B. E. *et al.* Parental education, parent–child relations and diagnosed mental disorders in childhood: prospective child cohort study. *Eur. J. Public Health* **31**, 514–520 (2021).
15. Bath, K. G. Synthesizing Views to Understand Sex Differences in Response to Early Life Adversity. *Trends Neurosci.* **43**, 300–310 (2020).
16. Rackerby, B., Kim, H. J., Dallas, D. C. & Park, S. H. Understanding the effects of dietary components on the gut microbiome and human health. *Food Sci. Biotechnol.* **29**, 1463–1474 (2020).
17. Korpela, K. *et al.* Intestinal microbiome is related to lifetime antibiotic use in Finnish pre-school children. *Nat. Commun.* **7**, 10410 (2016).
18. Uy, J. P. *et al.* Effects of maternal childhood trauma on child emotional health: maternal mental health and frontoamygdala pathways. *J. Child Psychol. Psychiatry* **64**, 426–436 (2023).
19. Querdasi, F. R. *et al.* Multigenerational adversity impacts on human gut microbiome composition and socioemotional functioning in early childhood. *Proc. Natl. Acad. Sci.* **120**, e2213768120 (2023).
20. Ta, L. D. H. *et al.* A compromised developmental trajectory of the infant gut microbiome and metabolome in atopic eczema. *Gut Microbes* **12**, 1–21 (2020).
21. Chen, L. W. *et al.* Implication of gut microbiota in the association between infant antibiotic exposure and childhood obesity and adiposity accumulation. *Int. J. Obes. 2020 447* **44**, 1508–1520 (2020).
22. Xu, J. *et al.* Ethnic diversity in infant gut microbiota is apparent before the introduction of complementary diets. *Gut Microbes* **11**, 1362–1373 (2020).

23. Caporaso, J. G. *et al.* Ultra-high-throughput microbial community analysis on the Illumina HiSeq and MiSeq platforms. *ISME J. 2012 68* **6**, 1621–1624 (2012).

24. Smith, S. M. *et al.* Advances in functional and structural MR image analysis and implementation as FSL. *NeuroImage* **23**, S208–S219 (2004).

25. Power, J. D. *et al.* Methods to detect, characterize, and remove motion artifact in resting state fMRI. *NeuroImage* **84**, 10.1016/j.neuroimage.2013.08.048 (2013).

26. Zhang, Y., Brady, M. & Smith, S. Segmentation of brain MR images through a hidden Markov random field model and the expectation-maximization algorithm. *IEEE Trans. Med. Imaging* **20**, 45–57 (2001).

27. Douglas, G. M. *et al.* PICRUSt2 for prediction of metagenome functions. *Nat. Biotechnol.* **38**, 685–688 (2020).

28. Nieminen, P. Application of Standardized Regression Coefficient in Meta-Analysis. *BioMedInformatics* **2**, 434–458 (2022).

29. Carlson, A. L. *et al.* Infant gut microbiome composition is associated with non-social fear behavior in a pilot study. *Nat. Commun. 2021 121* **12**, 1–16 (2021).

30. Albertina, E. A., Barch, D. M. & Karcher, N. R. Internalizing Symptoms and Adverse Childhood Experiences Associated With Functional Connectivity in a Middle Childhood Sample. *Biol. Psychiatry Cogn. Neurosci. Neuroimaging* **9**, 50–59 (2024).
