# Supplementary material for: Childhood gut microbiome is linked to internalizing symptoms at school age via the functional connectome
Source: Nat Commun. 2025 Oct 30;16:9359. doi: 10.1038/s41467-025-64988-6 (PMC12575631; doi:10.1038/s41467-025-64988-6)
Supplement: Supplementary file 2 — Reporting Summary [file 41467_2025_64988_MOESM2_ESM.pdf]

nature portfolio

Corresponding author(s): Francesca Querdasi

Last updated by author(s): 5/3/2025

Reporting Summary

Nature Portfolio wishes to improve the reproducibility of the work that we publish. This form provides structure for consistency and transparency in reporting. For further information on Nature Portfolio policies, see our Editorial Policies and the Editorial Policy Checklist.

Statistics

For all statistical analyses, confirm that the following items are present in the figure legend, table legend, main text, or Methods section.

n/a	Confirmed	
☐	☒	The exact sample size (*n*) for each experimental group/condition, given as a discrete number and unit of measurement
☐	☒	A statement on whether measurements were taken from distinct samples or whether the same sample was measured repeatedly
☐	☒	The statistical test(s) used AND whether they are one- or two-sided<br>*Only common tests should be described solely by name; describe more complex techniques in the Methods section.*
☐	☒	A description of all covariates tested
☐	☒	A description of any assumptions or corrections, such as tests of normality and adjustment for multiple comparisons
☐	☒	A full description of the statistical parameters including central tendency (e.g. means) or other basic estimates (e.g. regression coefficient) AND variation (e.g. standard deviation) or associated estimates of uncertainty (e.g. confidence intervals)
☐	☒	For null hypothesis testing, the test statistic (e.g. *F*, *t*, *r*) with confidence intervals, effect sizes, degrees of freedom and *P* value noted<br>*Give P values as exact values whenever suitable.*
☒	☐	For Bayesian analysis, information on the choice of priors and Markov chain Monte Carlo settings
☒	☐	For hierarchical and complex designs, identification of the appropriate level for tests and full reporting of outcomes
☐	☒	Estimates of effect sizes (e.g. Cohen's *d*, Pearson's *r*), indicating how they were calculated

Our web collection on statistics for biologists contains articles on many of the points above.

Software and code

Policy information about availability of computer code

Data collection	Custom code was not used for data collection.
Data analysis	Data analysis was performed with R v4.2.3. SPLS models were computed using the MixOmics package, and mediation models were run using the PROCESS macro for R. Metagenomic functional prediction was performed using PICRUSt v2.6.2. Custom code is publicly available at: https://github.com/fquerdasi/gusto_microbiome_brain_internalizingsx.

For manuscripts utilizing custom algorithms or software that are central to the research but not yet described in published literature, software must be made available to editors and reviewers. We strongly encourage code deposition in a community repository (e.g. GitHub). See the Nature Portfolio guidelines for submitting code & software for further information.

Data

Policy information about availability of data

All manuscripts must include a data availability statement. This statement should provide the following information, where applicable:

- Accession codes, unique identifiers, or web links for publicly available datasets
- A description of any restrictions on data availability
- For clinical datasets or third party data, please ensure that the statement adheres to our policy

Data from the GUSTO study are not publicly available due to multi-institutional cohort data governance and ethical restrictions. Access procedures modeled after those of the NIH can be used to obtain access to the data. After a request is submitted, it is reviewed and approved by the GUSTO Executive Committee (of which C.Y.S., P.D.G., K.G., and M.J.M. are members). Requests can be sent to Li Ting (ang_li_ting@sics.a-star.edu.sg) to initiate the data access submission-to-approval

workflow. Please allow 10 working days for a response.

Research involving human participants, their data, or biological material

Policy information about studies with human participants or human data. See also policy information about sex, gender (identity/presentation), and sexual orientation and race, ethnicity and racism.

Reporting on sex and gender	Information was collected on child sex via hospital records when children were born. Counts for each sex (male, female) in the analytic sample are included in Table 1 of the manuscript. We did not examine sex differences or sex interactions due to small sample size, and lack of targeted hypotheses surrounding sex differences on our research questions.
Reporting on race, ethnicity, or other socially relevant groupings	Mother self-reported their and their child's ethnicity during study recruitment. The GUSTO cohort study specifically recruited pregnant women who were of Chinese, Malay, or Indian ethnicity (the largest ethnic groups in Singapore), and who's child was also of homogenous ethnic background (i.e., both parents shared the same ethnicity). Counts for each ethnicity in our analytic sample are reported in Table 1.
Population characteristics	In addition to sex and ethnicity characteristics, children in the sample were born full-term (>36 weeks gestation), with roughly 70% being born via vaginal birth and the rest via cesarean section. See Table 1 in the manuscript for sample demographics and descriptive statistics on all relevant covariates.
Recruitment	The GUSTO study recruited pregnant citizens and permanent residents of Singapore during their antenatal dating ultrasound scan appointment in the first trimester of pregnancy at one of two major public maternity hospitals in Singapore between June 2009 and September 2010. As described above, children in the study were required to have a homogenous ethnic background including ethnicity from one of the 3 most common ethnic groups in Singapore, so the findings may not generalize to other ethnicities or multi-ethnic or minority Singaporean populations.
Ethics oversight	The GUSTO study was approved by the National Healthcare Group Domain Specific Review Board and SingHealth Centralized Institutional Review Board in Singapore.

Note that full information on the approval of the study protocol must also be provided in the manuscript.

Field-specific reporting

Please select the one below that is the best fit for your research. If you are not sure, read the appropriate sections before making your selection.

☐ Life sciences ☒ Behavioural & social sciences ☐ Ecological, evolutionary & environmental sciences

For a reference copy of the document with all sections, see nature.com/documents/nr-reporting-summary-flat.pdf

Behavioural & social sciences study design

All studies must disclose on these points even when the disclosure is negative.

Study description	This is an observational, longitudinal, quantitative study.
Research sample	The research sample included mothers and their children (58% female, 42% male) participating in the GUSTO study. Mothers and children were of homogenous ethnic background from one of the 3 most common ethnic groups in Singapore (Chinese, Malay,o r Indian). The sample was 73% Chinese, 18% Malay, and 9% Indian. This ethnic breakdown is close to representative of the larger Singaporean population, of which 74% identified as Chinese, 14% as Malay, 9% as Indian, and 3% as another ethnicity. Our sample thus has a slightly higher proportion of Malay participants and lacks representation of other ethnicities. The study sample includes dyads from the GUSTO cohort for whom the child donated a usable stool sample for gut microbiome analysis at age 2 years and contributed good quality resting-state functional magnetic resonance imaging brain data at age 6 years, and a caregiver reported on the child's internalizing symptoms at age 7.5 years.
Sampling strategy	Convenience sampling, recruiting pregnant women who attended their first trimester antenatal dating ultrasound at Singapore's two major public maternity hospitals, was used. We included all dyads from the GUSTO study who provided data on our key variables of interest (described above). Our obtained sample size of N=55 is sufficient for the exploratory, data-driven analysis method we used, which is robust to small sample size, and to generate initial findings to generate hypotheses for future studies in larger samples.
Data collection	Questionnaire and demographic data was collected via pen and paper or online form, depending on capacity of the research team at each timepoint of data collection (i.e., demographics collected during pregnancy, internalizing symptom assessment at age 7.5 years). No one was present for data collection besides the participant and researcher. Brain MRI data was collected by a 3 Tesla MRI scanner, and gut microbiome data was collected via stool sampling kits that were sent home with participants for them to complete in their own home and then return to the research team. This was an observational study so no experimental assignment or blinding was conducted.
Timing	Mother-child dyads were recruited in the first trimester of pregnancy at one of two major public maternity hospitals in Singapore between June 2009 and September 2010. Demographic information was collected during recruitment, stool samples were collected when children were 2 years of age, MRI data were collected when children were 6 years of age, and caregiver-reported assessment of internalizing symptoms was collected when children were 7.5 years of age.

Data exclusions	The current study includes 110 children from the GUSTO cohort who donated a usable stool sample for microbiome analysis at age 2 years, contributed resting-state functional magnetic resonance imaging (rs-fMRI) data at age 6 years, and whose caregiver reported on their internalizing symptoms at age 7.5 years, of which 55 had good-quality imaging data. Participants who did not provide usable data on the key variables of interest (i.e., stool sample, MRI brain scan, symptom assessment) were excluded. Exclusion criteria were pre-established.
Non-participation	The main GUSTO study began with 1,450 pregnant women enrolled. Of those, 442 dropped out between the pregnancy and 2 year timepoints, 55 dropped out between the 2 year and 6 year timepoints, and 10 dropped out between the 6 year and 7.5 year timepoints. Reasons for drop out included participants being lost to follow up, personal or family circumstances, and inconvenience of the study.
Randomization	Participants were not randomized into experimental groups.

Reporting for specific materials, systems and methods

We require information from authors about some types of materials, experimental systems and methods used in many studies. Here, indicate whether each material, system or method listed is relevant to your study. If you are not sure if a list item applies to your research, read the appropriate section before selecting a response.

Materials & experimental systems

n/a	Involved in the study
☒	☐ Antibodies
☒	☐ Eukaryotic cell lines
☒	☐ Palaeontology and archaeology
☒	☐ Animals and other organisms
☒	☐ Clinical data
☒	☐ Dual use research of concern
☒	☐ Plants

Methods

n/a	Involved in the study
☒	☐ ChIP-seq
☒	☐ Flow cytometry
☐	☒ MRI-based neuroimaging

Plants

Seed stocks	*Report on the source of all seed stocks or other plant material used. If applicable, state the seed stock centre and catalogue number. If plant specimens were collected from the field, describe the collection location, date and sampling procedures.*
Novel plant genotypes	*Describe the methods by which all novel plant genotypes were produced. This includes those generated by transgenic approaches, gene editing, chemical/radiation-based mutagenesis and hybridization. For transgenic lines, describe the transformation method, the number of independent lines analyzed and the generation upon which experiments were performed. For gene-edited lines, describe the editor used, the endogenous sequence targeted for editing, the targeting guide RNA sequence (if applicable) and how the editor was applied.*
Authentication	*Describe any authentication procedures for each seed stock used or novel genotype generated. Describe any experiments used to assess the effect of a mutation and, where applicable, how potential secondary effects (e.g. second site T-DNA insertions, mosiacism, off-target gene editing) were examined.*

Magnetic resonance imaging

Experimental design

Design type	Resting state
Design specifications	One resting state scan per subjct
Behavioral performance measures	No behavioral performance measures were recording for resting state scans.

Acquisition

Imaging type(s)	T1-weighted structural scan, functional scan
Field strength	3T
Sequence & imaging parameters	T1-weighted Magnetization Prepared Rapid Gradient Recalled Echo images (MPRAGE; 160 slices, 1 mm thickness, field-of-view = 192 × 192 mm2 , matrix = 192 × 192, repetition time = 2000 ms, echo time = 2.08 ms, inversion time = 877 ms, flip angle = 9◦, scanning time = 3.5 min) and resting state (rs) fMRI images (single-shot echo-planar imaging; 48 slices with 3 mm slice thickness, no interslice gaps, matrix = 64 × 64, field- of-view = 192×192 mm2, echo time = 27 ms, flip angle = 90◦, repetition time = 2.62–2.66 sec, scanning time = 5.27 min) were acquired
Area of acquisition	Whole brain scan

Diffusion MRI ☐ Used ☒ Not used

Preprocessing

Preprocessing software: We used FSL version 5.0.9 for all preprocessing steps.

Normalization: Linear registration from subject space to standardized space

Normalization template: MNI152 template

Noise and artifact removal: For functional images, preprocessing included temporal high-pass filtering using a 0.01 Hz filter, smoothing using a 6 mm full-width half maximum Gaussian kernel, and linear registration to the structural image (with 6 degrees of freedom).

We used fsl_motion_outliers to generate confound matrices to censor volumes that exceeded 0.2 mm framewise displacement (FD), which we included in the general linear model. We excluded scans that had greater than 36 (30%) frames exceeding 0.2 mm FD and/or any frames exceeding 2mm FD (N = 53 of N = 110 collected scans were excluded). We then visually inspected all rs-fMRI scans to ensure that the brain was sufficiently in view and there were no large-scale motion artifacts (no scans were excluded in this stage). Finally, for remaining subjects, we visually inspected each subjects' structural scan for the presence of motion artifacts and excluded scans with extensive artifacts (N = 2 additional scans excluded).

We used FSL's Automatic Segmentation Tool (FAST) to segment the structural image into gray matter, white matter, and cerebrospinal fluid masks. After transforming masks into each subjects' functional space, we extracted signals from those masks and included them as nuisance regressors along with their temporal derivatives to account for the global signal. We also included standard and extended motion parameters (24 parameters) as nuisance regressors.

Volume censoring: We used fsl_motion_outliers to generate confound matrices to censor volumes that exceeded 0.2 mm framewise displacement (FD), which we included in the general linear model.

Statistical modeling & inference

Model type and settings: General linear model. At level 1, we included confound matrices generated from fsl_motion_outliers to censor volumes that exceeded 0.2 mm framewise displacement (FD). We used FSL's Automatic Segmentation Tool (FAST) to segment the structural image into gray matter, white matter, and cerebrospinal fluid masks. After transforming masks into each subjects' functional space, we extracted signals from those masks and included them as nuisance regressors along with their temporal derivatives to account for the global signal. We also included standard and extended motion parameters (24 parameters) as nuisance regressors. Each participant only had one run.

Effect(s) tested: N/A; resting state scan

Specify type of analysis: ☐ Whole brain ☒ ROI-based ☐ Both

Anatomical location(s): Regions of interest (ROIs) were selected from the well-validated, functionally defined Seitzman 300-ROI parcellation atlas. Each ROI is assigned to one of 13 predefined networks: auditory (AUD), cingulo-opercular (CON), default mode (DMN), dorsal attention (DAN), frontal parietal (FPN), medial temporal lobe (MTL), parietomedial (PMN), striatal-orbitofrontal-amygdalar (SOFA), salience (SAL), dorsal somatomotor (SMD), lateral somatomotor (SML), ventral attention (VAN), and visual (VIS)

Statistic type for inference (See Eklund et al. 2016): N/A; we extracted time series data from each ROI and calculated connectivity metrics within and between networks.

Correction: We did not conduct voxel- or cluster-wise analyses. We used a data-driven, data reduction approach (sparse partial least squares), entering all derived intra- and inter-network connectivity values to identify connectivity patterns that explain significant variability (accounting for multicollinearity among connectivity values) in a behavioral outcome.

Models & analysis

n/a	Involved in the study
☐	☒ Functional and/or effective connectivity
☒	☐ Graph analysis
☐	☒ Multivariate modeling or predictive analysis

Functional and/or effective connectivity: For each participant, we extracted the time series of each ROI from the processed image and calculated RSFC strength using Fisher r-to-z transformation of the average correlation values between ROI-pairs within (n=13; i.e., intra-network connectivity) and between (n=78; i.e., inter-network connectivity) each network (e.g., DMN-FPN, DMN-CON).

Multivariate modeling and predictive analysis: We employed sparse partial least squares (sPLS) regression to identify linear combinations of brain networks at age 6 years (predictor dataset) that maximally covaried with internalizing symptoms at age 7.5 years (outcome variable) using the MixOmics R package. sPLS regression models were tuned for the optimal number of brain network components and variables per component using 10-fold cross validation repeated 100 times. The model that maximized R2 was selected as best-fitting. For each final tuned model, we report a

Pearson's correlation between scores on the derived signature from the final model with the outcome variable, the loading of each network onto each component (which measures the relative strength of each network's contribution and the direction of its association with internalizing symptoms), and the Variable Importance in the Projection (VIP) of each network onto each component (which represents the percent of variance in internalizing symptoms that is explained by the network divided by the total variance explained by the component). Next, we used sPLS regression to identify linear combinations of genera abundances (i.e., 'microbial profiles') at age 2 years (predictor dataset) that maximally covaried with the 'brain signatures' (outcome variable) identified in the previous analysis step, using the same tuning and evaluation steps as stated above.
